# Incidence, burden, and associated factors of severe potential drug–drug interactions in intensive care unit patients: a real-world retrospective study

**DOI:** 10.3389/fphar.2026.1920401

**Published:** 2026-09-24

**Authors:** Huaqiao Jiang, Tingting Shen, Yun Yang, Lin Zhang, Xiaofang Tan, Xiaoqun Lv, Yanhua Lin, Ning Zhang

**Affiliations:** 1 Department of Pharmacy, Jinshan Hospital, Fudan University, Shanghai, China; 2 Department of Dermatology, Jinshan Hospital, Fudan University, Shanghai, China; 3 Department of Nursing, Jinshan Hospital, Fudan University, Shanghai, China; 4 Department of Intensive Care, Jinshan Hospital, Fudan University, Shanghai, China

**Keywords:** central nervous system depression, intensive care unit, medication safety burden, polypharmacy, severe potential drug-drug interactions

## Abstract

**Introduction:**

Severe potential drug-drug interactions (pDDIs) in intensive care unit (ICU) patients remain insufficiently characterized. This study investigated the incidence, patterns, burden, and associated factors of severe pDDIs.

**Methods:**

This single-center retrospective cohort included ICU admissions from January 2023 through December 2024 for adults aged ≥18 years who received ≥2 eligible systemic medications administered orally or intravenously. Category D and X pDDIs were identified with Lexi-Interact (Lexidrug, UpToDate) using confirmed administration records. Logistic and ordinal logistic regression were performed to evaluate severe-pDDI occurrence (≥1) and admission-level burden (0, 1–2, or ≥3).

**Results:**

A total of 1,170 ICU admissions were included, involving 26,627 medication administrations. 64.44% were male and 55.38% received mechanical ventilation support. The most common implicated pharmacological group was alimentary-tract and metabolism agents, while ambroxol and glutathione emerged as the top two individual drugs. Among 1,170 ICU admissions, 1,092 (93.33%) exhibited pDDIs, and 769 (65.73%) experienced at least one category D or X pDDI. The 2,704 severe pDDIs comprised 1,681 (62.17%) category D and 1,023 (37.83%) category X events, yielding 231.1 pDDIs per 100 admissions and 101.6 pDDIs per 1,000 medication administrations. Nervous-system agents predominated; butorphanol [948/5,408 ingredient involvements (17.53%)] and dexmedetomidine [824/5,408 (15.24%)] were most frequently implicated. Butorphanol–dexmedetomidine [335/2,704 events (12.39%)] and noscapine–chlorphenamine [176/2,704 (6.51%)] were the leading category D and X pairs, respectively. Additive pharmacodynamic central nervous system depression predominated. Mechanical ventilation and medication count were the strongest adjusted correlates of severe-pDDI occurrence and burden, while baseline variables (sex, APACHE II score) lost significance after adjusting for ICU process variables. These associations remained materially unchanged after excluding 29 readmission encounters. In an exploratory review of 50 severe-pDDI episodes, 32 (64.0%) had temporally associated clinical signs or events documented and 29 (58.0%) had a documented management action.

**Conclusion:**

Category D or X pDDI alerts were frequent, primarily driven by pharmacodynamic additive central nervous system depression from sedative–analgesic combinations. Treatment intensity rather than baseline characteristics was associated with severe-pDDI exposure. Regional ICU-tailored surveillance and pharmacist-integrated medication management may facilitate identification of high-risk combinations, although specific drug pairs require validation across different healthcare settings.

## 1 Introduction

Potential drug-drug interactions (pDDIs) are defined as the simultaneous or sequential administration of two or more interacting drugs that may lead to clinically relevant outcomes such as treatment failure or life-threatening adverse drug reactions (ADRs) (Mathilde et al., 2021; Bettonte et al., 2022). Potential DDIs represent a critical medication safety concern, especially among intensive care unit (ICU) patients who are highly vulnerable due to polypharmacy, organ dysfunction, altered drug metabolism, and physiological changes during critical illness (Grace et al., 2019; Morales Castro D et al., 2023). Compared with general wards, ICUs therefore generate a particularly high volume of interaction alerts (Uijtendaal et al., 2014; Grace et al., 2019).

Severe or high-risk pDDIs are of particular clinical importance because they may necessitate proactive clinical intervention, such as therapy modification, enhanced monitoring, or avoidance of drug combinations. Nevertheless, database alerts are not equivalent to clinically manifest adverse drug events (ADEs): clinical adjudication often reduces the number of alerts that are deemed actionable (Bakker et al., 2021), although selected interactions have been associated with preventable harm and additional treatment burden (Uijtendaal et al., 2014; Klopotowska JE et al., 2024).

The mechanism of DDIs may be pharmacokinetic, through altered absorption, distribution, metabolism, or elimination, or pharmacodynamic, involving additive, synergistic, or antagonistic effects. Pharmacodynamic interactions are especially relevant in the ICU because sedatives, analgesics, and other central nervous system (CNS)-active agents are often co-administered, creating predictable risks of additive CNS depression and hemodynamic instability (Riera et al., 2022).

Numerous ICU studies consistently report frequent pDDIs, but estimates vary with case mix, exposure definitions, and interaction databases (Ali et al., 2020; Obeid and Karara, 2022; Wang et al., 2022). Studies comparing different interaction databases show appreciable heterogeneity (Bakker et al., 2022; Han et al., 2025), while analyses using actual medication administrations and clinical adjudication highlight the importance of confirmed exposure and patient context (Bakker et al., 2021). Sedatives, analgesics, anti-infectives, and cardiovascular drugs recur across settings, and medication count and treatment intensity are established correlates (Riera et al., 2022; Wang et al., 2022; Kayhan et al., 2025). Therapy-specific investigations have examined anti-infective interactions (Koeck et al., 2021; Jiang HY et al., 2023; Xu et al., 2025), while lung-transplant data further illustrate how treatment context changes the interaction spectrum (Zhang et al., 2024).

Accordingly, this study makes an incremental contribution rather than identifying a previously unstudied problem. Although previous studies have advanced the field, several important aspects remain insufficiently characterized. While prior studies have investigated the prevalence, characteristics, and associated factors of pDDIs among ICU patients, most have not specifically focused on the quantitative distribution of severe pDDIs requiring therapy modification or avoidance. Moreover, the patient-level distribution of severe-pDDI burden and the relative contribution of baseline characteristics versus ICU treatment-related factors remain incompletely understood. Furthermore, the patterns of frequently occurring severe pDDI combinations may vary across ICU settings and require characterization using real-world medication administration data. Therefore, a comprehensive evaluation of severe pDDIs based on real-world ICU medication administration data is warranted to better identify high-risk patients and inform locally tailored ICU medication-safety strategies.

To address these gaps, this study aimed to: (1) characterize the incidence, patterns and burden distribution of severe pDDIs (Lexi-Interact categories D and X) among ICU patients by employing Lexi-Interact software based on real-world medication administration data during hospitalization, and (2) identify factors associated with severe-pDDI occurrence and burden.

## 2 Materials and methods

### 2.1 Study design, setting and data source

This single-center retrospective observational cohort study was conducted in the 23-bed mixed adult ICU of Jinshan Hospital, a 1,000-bed comprehensive public tertiary hospital affiliated to Fudan University in Shanghai, China. ICU admissions from January 2023 through December 2024 were eligible.

### 2.2 Study population

Admissions were excluded according to the following criteria: (1) age <18 years; (2) fewer than two drugs administered during the ICU stay; (3) automatic discharge or death within 24 h of ICU admission without any executed medical orders; (4) incomplete records or unclear medication documentation; (5) other situations not meeting the inclusion criteria. Of 1,238 admissions screened, exclusions were age <18 years (n = 15), ICU stay ≤24 h (n = 50), and incomplete or unclear records (n = 3); no admission was excluded solely for receiving fewer than two eligible medications (n = 0). Each ICU readmission was counted as a separate encounter; 29/1,170 included encounters (2.48%) were readmissions. Primary analyses treated encounters as independent.

### 2.3 Data collection

The demographic and clinical characteristics, including sex, age, body mass index (BMI), smoking and alcohol-use history, primary diagnosis at ICU admission, mechanical ventilation (MV) status, Acute Physiology and Chronic Health Evaluation (APACHE) II score (a validated tool for assessing disease severity), ICU length of stay, and patient outcomes were collected. A comprehensive medication profile was documented for each patient, including all medications administered during the ICU stay with their name, dose, route of administration, and start/end times for each administration. In this study, “medications” referred to those actually administered and recorded in the hospital charge system and nursing drug administration records (i.e., executed and charged), rather than those merely prescribed in electronic health records. If the time interval between administrations of the same medication was ≤24 h, the separate administrations were combined into one medication administration record. The start time of the first administration and the stop time of the last administration were recorded. Diagnoses were classified according to the International Classification of Diseases (ICD-10 codes), and drugs were grouped by the World Health Organization Anatomical Therapeutic Chemical (WHO-ATC) Classification (WHO, 2025). All extracted demographic, clinical, and medication-related data were independently reviewed by two clinically experienced investigators for quality control. Any discrepancies in data extraction or classification were discussed and resolved according to predefined criteria. Because this was verification of rule-based coding rather than independent blinded clinical adjudication, an inter-rater agreement statistic was not calculated.

### 2.4 Identification of severe pDDIs

Potential DDIs were identified using Lexi-Interact within UpToDate Lexidrug (Wolters Kluwer Clinical Drug Information, Inc., Hudson, OH, United States; accessed in 2025) and were divided into five risk levels: X (avoid combination therapy), D (consider therapy modification), C (monitor therapy), B (no action needed), and A (no known interaction). Lexi-Interact is a widely recognized drug and herbal interaction analysis tool capable of evaluating pDDIs and drug–allergy interactions (Grace et al., 2019; Han et al., 2025). Category D interactions are more severe than category C or B interactions and typically necessitate specific clinical interventions (e.g., alternative therapies). Category X interactions are contraindicated, as risks clearly outweigh benefits. Categories D and X were prespecified as severe pDDI alerts because both require active clinical consideration; pooling facilitated estimation of overall alert burden but did not imply equivalent urgency or observed clinical harm. Database classification followed Lexi-Interact criteria without investigator clinical adjudication. Additionally, two outcomes that the presence of at least one severe pDDI during the ICU stay (binary outcome) and the admission-level number of severe pDDIs (count outcome) were assessed. Topical and inhaled preparations, maintenance electrolyte and nutritional solutions, vaccines, temporarily or intermittently administered medications, and agents not searchable in Lexi-Interact during the study period were excluded, and only systemically administered (including oral or intravenous administration) drugs were analyzed. Concurrent exposure was defined as overlapping administration exceeding 24 h. However, the transition from one drug to another was not considered as co-administration. Combination products were assessed according to their active ingredients.

### 2.5 Burden analysis of severe pDDIs

In this study, “Severe-pDDI burden” was defined as a study-specific admission-level operational measure: the number of category D and X pDDI alerts during an ICU encounter, rather than a population-level disease burden metric. It was categorized as 0 (none), 1–2 (low burden), or ≥3 alerts (higher burden). Because no validated clinical threshold exists, these empirically selected categories describe no, lower, and relatively higher alert counts and should be interpreted as exploratory rather than validated clinical risk strata. Associations were evaluated with ordinal logistic regression using a proportional-odds model. The proportional-odds assumption was assessed employing the test of parallel lines, and if violated, multinomial logistic regression was performed instead. A sensitivity analysis used an alternative classification of 0, 1, or ≥2 alerts.

### 2.6 Exploratory chart review of severe pDDIs

To further explore the clinical relevance of severe pDDIs, an exploratory chart review was conducted. Fifty severe-pDDI episodes were selected using a stratified random sampling approach based on Lexi-Interact severity categories (D and X). The sampling procedure was performed using a computer-generated random number sequence to ensure randomness and reproducibility. A clinical pharmacist performed the initial review of electronic medical records for the selected episodes, and a second clinical pharmacist reviewed the extracted clinical information for verification.

The assessment focused on documented clinical consequences and management actions temporally associated with severe pDDIs, including excessive sedation, respiratory depression, hypotension, bradycardia, arrhythmia/QT prolongation, enhanced monitoring, dose adjustment, drug discontinuation, and drug substitution. Multiple clinical consequences or management actions could be recorded for each severe pDDI episode when applicable. Due to the retrospective design and potential incompleteness of clinical documentation, causal relationships between pDDIs and ADEs were not assessed.

### 2.7 Statistical analysis

Continuous variables are reported as mean ± standard deviation or median (interquartile range [IQR]), as appropriate, and categorical variables as n (%). Group comparisons used Fisher’s exact test or Pearson’s χ^2^-test for categorical variables and the Student’s t-test or the Mann-Whitney U test for continuous variables. Binary logistic regression evaluated severe-pDDI occurrence, and proportional-odds logistic regression evaluated burden. Candidate variables were prespecified from clinical relevance and prior evidence and were entered in two stages: Model 1 included baseline variables (available at ICU admission: age, sex, BMI, APACHE II score, diagnosis count, and comorbidities), and Model 2 additionally included ICU process variables (obtained during ICU stay: MV and medication count), with the latter being included in the extended model. All eligible admissions during the study period were analyzed and *a priori* power calculation was not performed. The final cohort had complete data for variables in the regression models, so complete-case analysis was used without imputation. Because group comparisons and secondary associations were exploratory, multiplicity adjustment was not applied; effect estimates, 95% confidence intervals (CIs), consistency across models, and p values were interpreted together.

Multicollinearity was assessed with variance inflation factors (VIFs), using >5 as the prespecified threshold. Because ICU length of stay (LOS) could influence cumulative exposure, additional sensitivity models adjusted for LOS. Binary-model fit was assessed by Nagelkerke R^2^, the Hosmer–Lemeshow test, and area under the receiver operating characteristic curve (AUC), while the parallel-lines test and pseudo-R^2^ was described for ordinal-model fit. Main analyses treated admissions as independent. To assess the potential influence of repeat encounters, a sensitivity analysis excluded the 29 readmission encounters and re-estimated the binary and ordinal regression models. Statistical analysis was performed using IBM SPSS Statistics for Windows, version 26.0 (IBM Corp., Armonk, NY, United States). A p-value <0.05 was considered statistically significant.

## 3 Results

### 3.1 Demographic and clinical characteristics of the study population

Between January 2023 and December 2024, 1,238 ICU admissions were screened and 1,170 were eligible after applying the prespecified exclusion criteria for pDDI analysis (Figure 1). The cohort included 29 readmission encounters (2.48%). The cohort had a median age of 73 years (IQR 63–82) and 754 admissions (64.44%) involved male patients. The mean APACHE II score was 19.82 ± 7.29 and the median number of diagnoses was 8 (IQR 5–11). Respiratory system diseases were the leading ICD-10 category of primary admission diagnoses [272 (23.25%) of all admissions], 55.38% of admissions received MV support, and the median ICU LOS was 6.5 days (IQR 3.5–13.0) (Table 1).

**FIGURE 1:**
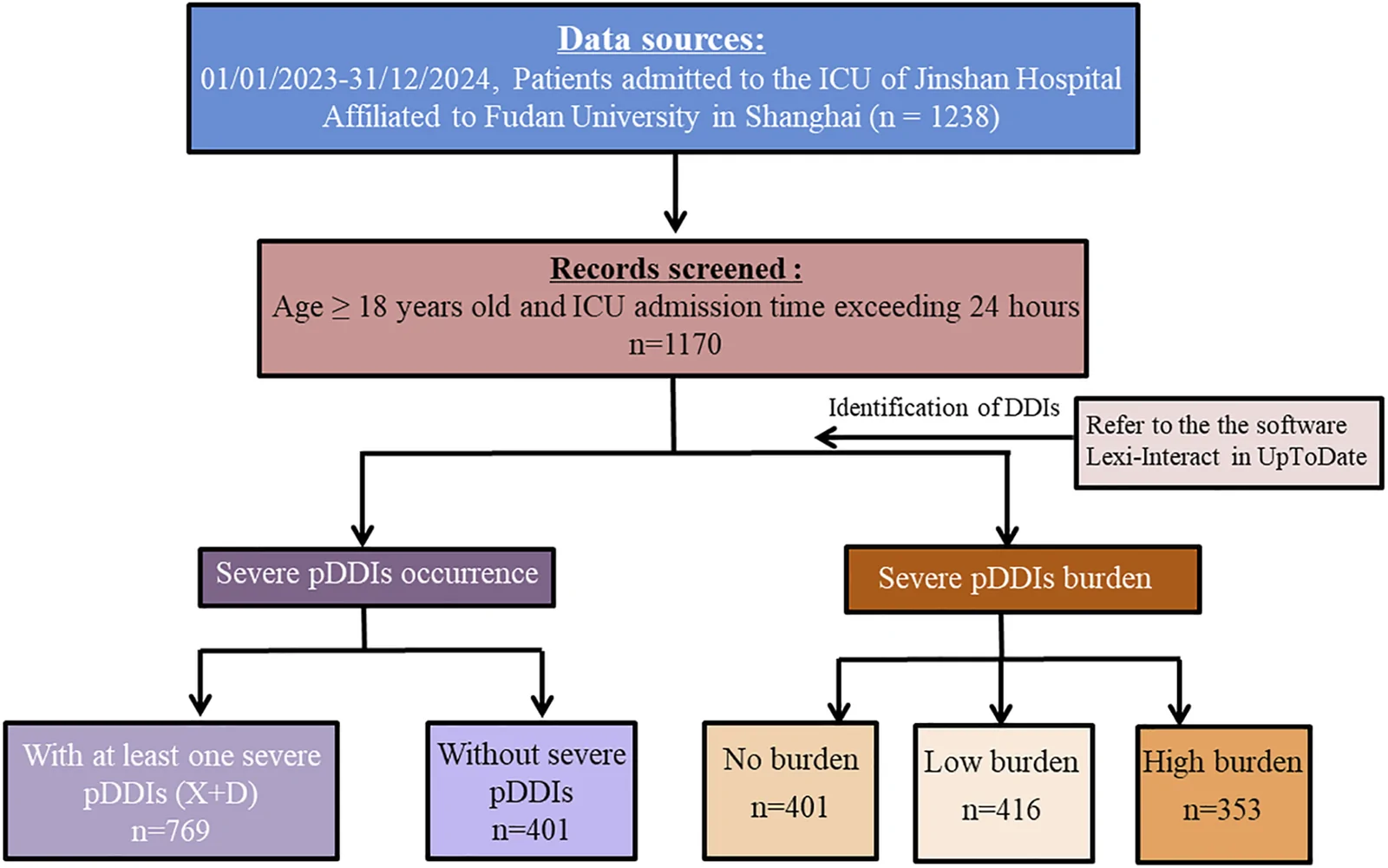
Flowchart depicting the study process. ICU, intensive care unit; pDDI, potential drug–drug interaction. Category D indicates consideration of therapy modification, and category X indicates avoidance of the combination.

**TABLE 1:** Characterization of ICU admissions by severe-pDDI occurrence.

Category	Total (N = 1,170, %)	Severe-pDDI occurrence		P value
		Yes (N = 769, %)	No (N = 401, %)	
Age, years, median (IQR)	73 (63–82)	74 (64–82)	72 (60–82)	0.189
Sex	​	​	​	0.001
Male	754 (64.44)	521 (67.75)	233 (58.10)	​
Female	416 (35.56)	248 (32.25)	168 (41.90)	​
BMI (kg/m^2^), median (IQR)	22.9 (20.0–25.8)	23.0 (20.2–26.0)	22.5 (19.9–25.4)	0.036
APACHE II score, mean	19.82 ± 7.29	20.40 ± 7.16	18.72 ± 7.42	<0.001
Number of diagnoses, mean	8.39 ± 4.15	8.40 ± 4.19	8.38 ± 4.07	0.929
Hypertension, n (%)	675 (57.69)	449 (58.39)	226 (56.36)	0.505
Diabetes, n (%)	329 (28.12)	204 (26.53)	125 (31.17)	0.094
Coronary heart disease, n (%)	538 (45.98)	370 (48.11)	168 (41.90)	0.043
Stroke, n (%)	400 (34.19)	258 (33.55)	142 (35.41)	0.524
Smoking, n (%)	214 (18.29)	150 (19.51)	64 (15.96)	0.136
Alcohol use, n (%)	142 (12.14)	96 (12.48)	46 (11.47)	0.615
Primary diagnosis category (ICD-10), n (%)	​	​	​	<0.001
Diseases of the respiratory system	272 (23.25)	221 (28.74)	51 (12.72)	​
Diseases of the circulatory system	222 (18.97)	160 (20.81)	62 (15.46)	​
Certain infectious and parasitic diseases	177 (15.13)	88 (11.44)	89 (22.19)	​
Injury, poisoning and certain other consequences of external causes	146 (12.48)	94 (12.22)	52 (12.97)	​
Diseases of the digestive system	113 (9.66)	54 (7.02)	59 (14.71)	​
Others*	240 (20.51)	152 (19.77)	88 (21.95)	​
Mechanical ventilation, n (%)	​	​	​	<0.001
Yes	648 (55.38)	514 (66.84)	134 (33.42)	​
No	522 (44.62)	255 (33.16)	267 (66.58)	​
Number of ICU medications, median (IQR)	21 (16–27)	23 (15–30)	17 (11–21)	<0.001
ICU LOS, days, median (IQR)	6.5 (3.5–13.0)	8 (4–15)	5 (3–8)	<0.001
ICU mortality, n (%)	116 (9.91)	88 (11.44)	28 (6.98)	0.015
Severe-pDDI count (D + X), median (IQR)	1 (0–3)	2 (1–4)	0	<0.001

* “Others” encompasses the remaining part of the ICD-10, classification system excluding the above-mentioned five systems; BMI, body mass index; ICU LOS, length of stay in the intensive care unit; IQR, interquartile range. p values compare admissions with versus without a severe pDDI.

Across all admissions, 26,543 medication records, which contained 497 different active pharmaceutical ingredients and 26,627 administrations in total, were administered, with a median of 21 medications per admissions (IQR 16–27). As shown in Table 2, the most frequently administered pharmacological groups by administration count were alimentary-tract and metabolism [7,171/26,627 (26.93%)], respiratory system [4,313/26,627 (16.20%)], cardiovascular system [3,856/26,627 (14.48%)], blood and blood forming organs [3,142/26,627 (11.80%)], and systemic antimicrobial drugs [2,938/26,627 (11.03%)]. However, the two most frequently administered individual drugs were ambroxol and glutathione, which belonged to the respiratory system and various ATC groups, respectively (Figure 2A).

**TABLE 2:** Quantities and ATC classifications of routinely administered medications and severe pDDI-involved medications in the ICU.

Drug class	Number of drugs	Frequency of drugs (%)	Frequency of severe pDDIs (%)	Frequency of top 20 severe pDDIs (%)
Alimentary tract and metabolism	90	7,171 (26.93)	473 (8.75)	4 (8.70)
Respiratory system	36	4,313 (16.20)	1,052 (19.45)	11 (23.91)
Cardiovascular system	79	3,856 (14.48)	323 (5.97)	3 (6.52)
Blood and blood forming organs	60	3,142 (11.80)	238 (4.40)	2 (4.35)
Antiinfectives for systemic use	71	2,938 (11.03)	390 (7.21)	2 (4.35)
Nervous system	42	1933 (7.26)	2,761 (51.05)	23 (50.00)
Various	25	1923 (7.22)	118 (2.18)	1 (2.17)
Systemic hormonal preparations, excl. Sex hormones and insulins	15	662 (2.49)	14 (0.26)	0
Traditional Chinese medicine	27	218 (0.82)	0	0
Dermatologicals	19	167 (0.63)	9 (0.17)	0
Sensory organs	9	114 (0.43)	0	0
Antiparasitic products, insecticides and repellents	3	66 (0.25)	0	0
Antineoplastic and immunomodulating agents	8	64 (0.24)	11 (0.20)	0
Musculo-skeletal system	9	32 (0.12)	11 (0.20)	0
Genito urinary system and sex hormones	4	28 (0.11)	8 (0.17)	0
Sum^※^	​	26,627 (100%)	​	​

※As certain medications were administered multiple times to individual patients, the total frequency of drug administrations exceeds total number of included patients.

**FIGURE 2:**
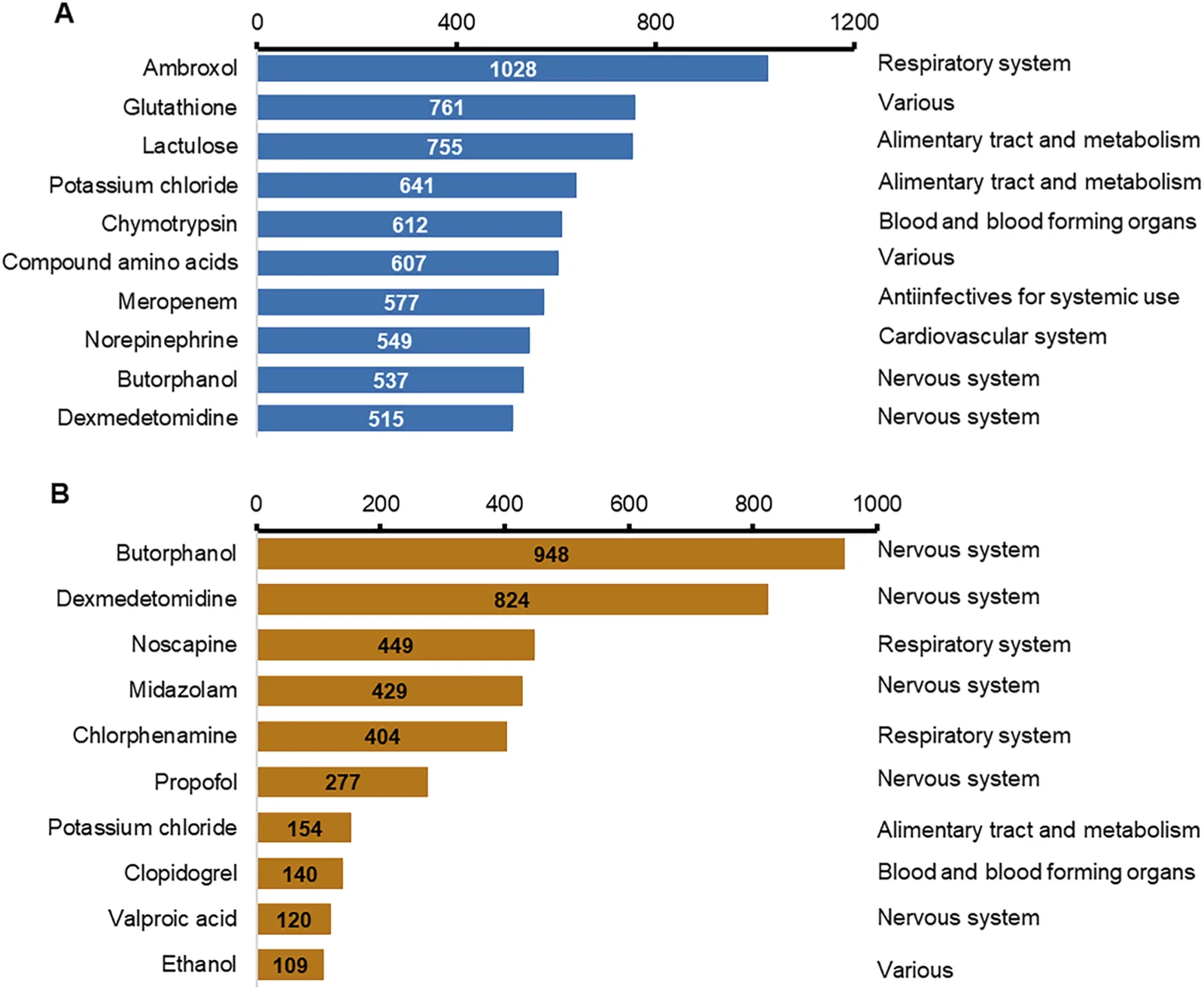
Top 10 medications by administration count and top 10 active ingredients by severe-pDDI involvement count in the ICU. **(A)** The 10 medications with the highest administration counts among 26,627 medication administrations. **(B)** The 10 active ingredients with the highest involvement counts among 5,408 ingredient involvements arising from 2,704 severe-pDDI events. Each severe-pDDI event contributed two ingredient involvements. The corresponding first-level ATC groups are shown on the right of each panel. Panels A and B use different denominators and independently scaled x-axes; therefore, bar lengths should not be compared directly between panels.

### 3.2 Primary outcome: the incidence and patterns of severe pDDIs (D + X)

Overall, 1,092 admissions (93.33% of all admissions) experienced at least one pDDIs during their ICU stay, and 13,789 pDDI events were identified; whereas 78 admissions had none (Counts of admissions and events by risk category are reported in Supplementary Table S1). However, severe pDDIs (categories D + X) occurred in 769 admissions (65.73% of all admissions), and 401 admissions (34.27%) had no severe pDDIs. Among the 769 affected admissions, there were 2,704 severe pDDIs involving 213 drug combinations that 1,023 were level X in 429 admissions, and 1,681 were level D in 637 admissions (Supplementary Table S2). Category D and X pDDIs account for 12.19% and 7.42% of all identified pDDIs, respectively. The maximum number of severe pDDIs (D + X) observed in a single admission was 23.

Table 3 presents pairs ranked 1–20, including ties (23 pairs). Butorphanol–dexmedetomidine [335/2,704 events (12.39%)] and noscapine–chlorphenamine [176/2,704 (6.51%)] were the leading category D and X pairs, respectively. Across the 5,408 ingredient involvements contributed by 2,704 severe pDDI events, nervous-system agents accounted for 2,761 (51.05%) and respiratory-system agents for 1,052 (19.45%). The 23 tied top-ranked pairs contributed 46 ingredient occurrences, of which 23 (50.00%) were nervous-system and 11 (23.91%) respiratory-system agents. The detailed ATC groups involved in severe pDDIs are listed in Table 2. Butorphanol, dexmedetomidine, noscapine, midazolam, and chlorphenamine were the active ingredients most frequently implicated in severe pDDIs (Figure 2B). Noscapine and chlorphenamine were components of compound methoxyphenamine products rather than single-ingredient medications. Additionally, additive pharmacodynamic (PD) mechanism predominated both overall and among the top 20 severe-pDDI pairs, and the most common potential clinical outcome was an increased risk of CNS depression.

**TABLE 3:** Top 20 most common severe-pDDI drug combinations.

Ranking	Risk rating	Drug pairs	Mechanism of interaction	Potential clinical consequences	Evidence	Severity	Frequency (%, total = 2,704)
1	D	Butorphanol-dexmedetomidine	PD	Increase CNS depressant effects	Intermediate	Major	335 (12.39%)
2	D	Butorphanol-midazolam	PD	Increase CNS depressant effects	Intermediate	Major	191 (7.06%)
3	X	Noscapine-chlorphenamine	PD	Increase adverse/toxic effects of noscapine	Intermediate-low	Moderate	176 (6.51%)
4	D	Butorphanol-propofol	PD	Increase CNS depressant effects	Intermediate	Major	122 (4.51%)
5	D	Dexmedetomidine-midazolam	PD	Increase CNS depressant effects	Intermediate-low	Moderate	118 (4.36%)
6	X	Doxofylline-aminophylline	PD	Increase adverse/toxic effects of doxofylline	Intermediate-low	Moderate	91 (3.37%)
7	D	Dexmedetomidine-chlorphenamine	PD	Increase CNS depressant effects	Intermediate-low	Moderate	84 (3.11%)
8	X	Noscapine-dexmedetomidine	PD	Increase adverse/toxic effects of noscapine	Intermediate-low	Moderate	83 (3.07%)
9	D	Dexmedetomidine-propofol	PD	Increase CNS depressant effects	Intermediate-low	Moderate	82 (3.03%)
10	X	Esomeprazole-clopidogrel	PK/PD	Decrease active metabolite exposure and antiplatelet effects of clopidogrel	Intermediate-low	Major	70 (2.59%)
	X	Spironolactone-potassium chloride	PD	Increase hyperkalemic effects	Intermediate	Major	70 (2.59%)
11	X	Potassium chloride-chlorphenamine	PD	Increase the risk of ulcers with potassium chloride	Intermediate-low	Moderate	69 (2.55%)
12	X	Noscapine-butorphanol	PD	Increase adverse/toxic effects of noscapine	Intermediate-low	Moderate	68 (2.51%)
	D	Butorphanol-chlorphenamine	PD	Increase CNS depressant effects	Intermediate	Major	68 (2.51%)
13	X	Ethanol-butorphanol	PD	Increase CNS depressant effects	Intermediate	Major	59 (2.18%)
14	X	Omeprazole-clopidogrel	PK/PD	Decrease antiplatelet effects and active metabolite exposure of clopidogrel	Intermediate	Major	51 (1.89%)
15	X	Noscapine-midazolam	PD	Increase adverse/toxic effects of noscapine	Intermediate-low	Moderate	45 (1.66%)
16	D	Butorphanol-valproic acid	PD	Increase CNS depressant effects	Intermediate	Major	41 (1.52%)
17	D	Bisoprolol-dexmedetomidine	PD	Increase rebound hypertensive and AV-blocking effects	Intermediate-low	Moderate	38 (1.41%)
18	D	Linezolid-norepinephrine	PD	Increase hypertensive effects	Intermediate	Moderate	32 (1.18%)
19	X	Noscapine-propofol	PD	Increase adverse/toxic effects of noscapine	Intermediate-low	Moderate	30 (1.11%)
20	X	Butorphanol-linezolid	PD	Increase serotonin syndrome effects	Intermediate-low	Moderate	29 (1.07%)
	D	Dexmedetomidine-valproic acid	PD	Increase CNS depressant effects	Intermediate-low	Moderate	29 (1.07%)

PD, pharmacodynamics; PK, pharmacokinetics; CNS, central nervous system; AV-blocking, Atrioventricular Block.

In unadjusted comparisons, MV, ICU medication count, APACHE II score (all with p < 0.001), male sex (p = 0.001), BMI (p = 0.036), and coronary heart disease (p = 0.043) were significantly associated with severe-pDDI occurrence (Table 4). No association was found between severe pDDIs and age, diagnosis count, hypertension, diabetes, prior stroke, smoking, or alcohol use (all with p > 0.05). In multivariate logistic regression, Model 1 (baseline variables) showed that male sex (OR 1.51, 95% CI 1.16–1.96, p = 0.002) and APACHE II score (per-point OR 1.03, 95% CI 1.01–1.05, p < 0.001) were associated with severe-pDDI occurrence. After ICU process variables were added (Model 2), these associations attenuated (sex OR 1.19, 95% CI 0.88–1.60, p = 0.260; APACHE II score OR 1.00, 95% CI 0.98–1.02, p = 0.821), whereas MV (OR 2.43, 95% CI 1.80–3.28, p < 0.001) and medication count (per-medication OR 1.15, 95% CI 1.12–1.18, p < 0.001) emerged as the strongest adjusted correlates. Although MV and medication count were moderately correlated (Pearson r = 0.337; p < 0.001), but all VIFs were <2. Hypertension, diabetes and coronary heart disease were not independently associated with severe pDDIs in either model (all with p > 0.05), whereas diagnosis count (OR 0.92, 95% CI 0.89–0.96, p < 0.001) and prior stroke (OR 0.70, 95% CI 0.51–0.95, p = 0.023) showed inverse associations in Model 2.

**TABLE 4:** Factors associated with severe-pDDI occurrence and burden: univariable and multivariable analyses.

Candidate factors	Primary outcome							Secondary outcome: 0, 1–2, or ≥3 severe pDDIs			
	Bivariate analysis			Multivariate analysis				Multivariate analysis			
	With ≥1 severe pDDIs (N = 769)	Without severe pDDIs (N = 401)	p value	Model 1		Model 2		Model 1		Model 2	
				OR (95%CI)	p value	OR (95%CI)	p value	OR (95%CI)	p value	OR (95%CI)	p value
Baseline variables											
Age, years, median (IQR)^a^	74 (64–82)	72 (60–82)	0.189	1.00 (1.00–1.01)	0.427	1.00 0.99–1.01)	0.594	1.00 (1.00–1.01)	0.455	1.00 (0.99–1.01)	0.474
Sex, n (male/female)	521/248	233/168	0.001	1.51 (1.16–1.96)	0.002	1.19 (0.88–1.60)	0.260	1.51 (1.19–1.90)	0.001	1.20 (0.93–1.55)	0.161
BMI (kg/m^2^)^a^	23. 0 (20.2–26.0)	22.5 (19.9–25.4)	0.036	1.00 (0.99–1.00)	0.541	1.00 (0.99–1.01)	0.995	1.00 (0.99–1.01)	0.654	1.00 (0.99–1.01)	0.755
APACHE II score^b^	20.40 ± 7.16	18.72 ± 7.42	<0.001	1.03 (1.01–1.05)	<0.001	1.00 (0.98–1.02)	0.821	1.03 (1.01–1.04)	<0.001	0.99 (0.97–1.01)	0.291
Number of diagnoses^b^	8.40 ± 4.19	8.38 ± 4.07	0.929	0.98 (0.95–1.02)	0.341	0.92 (0.89–0.96)	<0.001	1.00 (0.98–1.03)	0.791	0.94 (0.91–0.97)	<0.001
Hypertension	449	226	0.505	1.05 (0.81–1.38)	0.687	1.01 (0.75–1.36)	0.948	1.11 (0.88–1.39)	0.384	1.03 (0.81–1.33)	0.789
Diabetes	204	125	0.094	0.76 (0.57–1.01)	0.058	0.75 (0.54–1.04)	0.082	0.85 (0.66–1.09)	0.193	0.81 (0.62–1.07)	0.141
CHD	370	168	0.043	1.28 (0.97–1.70)	0.082	1.11 (0.80–1.52)	0.537	1.20 (0.94–1.53)	0.135	0.94 (0.72–1.23)	0.674
Stroke	258	142	0.524	0.82 (0.63–1.08)	0.156	0.70 (0.51–0.95)	0.023	0.81 (0.64–1.03)	0.084	0.70 (0.54–0.91)	0.007
Smoking	150	64	0.136	1.24 (0.80–1.91)	0.344	1.22 (0.75–2.01)	0.422	1.41 (0.98–2.04)	0.066	1.57 (1.05–2.33)	0.028
Alcohol use	96	46	0.615	0.86 (0.52–1.43)	0.557	0.83 (0.47–1.48)	0.531	0.76 (0.50–1.17)	0.216	0.63 (0.40–1.01)	0.057
Process variable											
MV, n (yes/no)	514/255	134/267	<0.001	​	​	2.43 (1.80–3.28)	<0.001	​	​	2.46 (1.89–3.17)	<0.001
Number of ICU medications^a^	23 (15–30)	17 (11–21)	<0.001	​	​	1.15 (1.12–1.18)	<0.001	​	​	1.17 (1.14–1.19)	<0.001

Model 1 included baseline variables available at admission. Model 2 additionally included MV, and ICU, medication count. Candidate variables were selected based on clinical relevance rather than solely statistical significance from univariable analyses. Females and the absence of corresponding comorbidities/events (e.g., absence of hypertension, diabetes, etc.) served as the reference groups for binary variables. ORs, for continuous variables are per one-unit increase. The P-values for the parallel-lines test in Model 1 and Model 2 (secondary outcome) were 0.219 and 0.505, respectively (p > 0.05), confirming model adequacy. Model-fit statistics are reported in Supplementary Table S6.

CI, confidence interval; IQR, interquartile range; OR, odds ratio; pDDIs, potential drug–drug interactions; MV, mechanical ventilation; CHD, coronary heart disease; APACHE II, score, Acute Physiology and Chronic Health Evaluation II, score.

^a^ Mann–Whitney U test.

^b^ Student’s T-test. All other baseline comparisons were performed using the Chi-square test.

### 3.3 Secondary outcome: the burden and distribution of severe pDDIs (D + X)

Among all admissions, 416 (35.56%) had 1–2 severe pDDIs, 353 (30.17%) had ≥3, and 401 (34.27%) had none. The 2,704 severe pDDIs identified in this cohort corresponded to 231.1 severe pDDIs per 100 ICU admissions and 101.6 severe pDDIs per 1,000 medication administrations. The median number of severe pDDIs was 1 (IQR 0–3) overall and 2 (IQR 1–4) among affected admissions (Table 1). When the burden was further stratified, admissions with 1, 2–4, and ≥5 severe pDDIs accounted for 23.85%, 25.98%, and 15.90% of the cohort, respectively. Subgroup analysis also revealed heterogeneity in burden distribution. The median number of severe pDDIs was 0 (IQR 0–1) in non-mechanically ventilated patients and 2 (IQR 1–4.75) in mechanically ventilated patients. When stratified by severity category, category D interactions accounted for 1,681 events, and category X interactions accounted for 1,023 events, corresponding to 63.1 and 38.4 severe events per 1,000 medication administrations, respectively. Overall, category D interactions contributed the larger proportion of the burden (62.17% of all severe pDDIs), whereas category X interactions still represented more than one-third.

The proportional-odds assumption was supported for ordinal Model 1 (p = 0.219) and Model 2 (p = 0.505). In multivariate logistic regression, baseline variables (Model 1) showed that male sex (OR 1.51, 95% CI 1.19–1.90, p = 0.001) and APACHE II score (OR 1.03, 95% CI 1.01–1.04, p < 0.001) were associated with a higher severe-pDDI burden. The addition of ICU process variables (Model 2) showed that MV (OR 2.46, 95% CI 1.89–3.17; p < 0.001) and medication count (OR 1.17, 95% CI 1.14–1.19; p < 0.001) were also associated with higher alert burden; all VIFs were <2 and the correlation of MV and medication count was r = 0.632 (p < 0.001) (Table 4). Smoking history was associated with burden (OR 1.57, 95% CI 1.05–2.33; p = 0.028) but not binary occurrence, so this secondary result was treated as exploratory and should therefore be interpreted cautiously. MV and medication count remained associated in the alternative 0/1/≥2 burden analysis (Supplementary Table S3). After additional adjustment for ICU LOS, LOS itself was not associated with binary occurrence (OR 1.01, 95% CI 0.98–1.04; p = 0.615) or ordinal burden (OR 1.00, 95% CI 0.98–1.02; p = 0.748) (Supplementary Table S4). In the sensitivity analysis excluding the 29 readmission encounters (N = 1,141), the associations of MV and ICU medication count remained materially unchanged for both severe-pDDI occurrence (MV: OR 2.46, 95% CI 1.81–3.33; medication count: OR 1.15, 95% CI 1.12–1.18; both p < 0.001) and burden (MV: OR 2.52, 95% CI 1.94–3.27; medication count: OR 1.17, 95% CI 1.14–1.19; both p < 0.001) (Supplementary Table S5). Binary Model 1 had limited discrimination (AUC 0.603), whereas Model 2 performed better (AUC 0.792); Nagelkerke R^2^ increased from 0.043 to 0.313. Similarly, ordinal pseudo-R^2^ increased from 0.041 to 0.390 (Supplementary Table S6).

### 3.4 Exploratory chart review of severe pDDIs

Among the 50 randomly selected severe-pDDI episodes (31 category D and 19 category X), 32 (64.0%) had at least one documented clinical consequence. A total of 42 documented clinical consequence events were identified. Respiratory depression was the most frequently documented consequence (22/50, 44.0%), followed by hypotension (9/50, 18.0%) and excessive sedation (7/50, 14.0%). Because individual severe pDDI episodes could be associated with multiple clinical manifestations, clinical consequence event counts should not be summed as unique events. These observations were not causally adjudicated.

At least one management action was documented in 29 (58.0%) episodes, including enhanced monitoring (14/50, 28.0%), dose adjustment (9/50, 18.0%), drug discontinuation (4/50, 8.0%), and drug substitution (2/50, 4.0%). Detailed findings from the chart review are presented in Table 5.

**TABLE 5:** Documentation identified during exploratory chart review of 50 severe-pDDI episodes.

Variable	n (%)
Reviewed episodes	50
D category	31 (62.0%)
X category	19 (38.0%)
Documented clinical consequences	
Variable	n (%)
Any documented clinical consequence	32 (64.00%)
Respiratory depression	22 (44.00%)
Hypotension	9 (18.00%)
Excessive sedation	7 (14.00%)
Bradycardia	3 (6.00%)
Arrhythmia/QT prolongation	1 (2.00%)
Bleeding	0
Electrolyte abnormality	0
Other documented consequence	15 (30.00%)
Documented management actions	
Variable	n (%)
Any documented management action	29 (58.00%)
Enhanced monitoring	14 (28.00%)
Dose adjustment	9 (18.00%)
Drug discontinuation	4 (8.00%)
Drug substitution	2 (4.00%)
No documented action	21 (42.00%)

Multiple clinical manifestations or management actions could be documented for one episode. Findings were temporally associated with the indexed pDDI, episode but were not causally adjudicated as adverse drug events.

## 4 Discussion

This retrospective study provides a granular analysis of the incidence, patterns, burden distribution and associated factors of severe pDDIs in a large cohort of 1,170 ICU admissions admitted to a comprehensive public tertiary hospital using real-world medication administration data. In this cohort, severe-pDDI alerts classified as Lexi-Interact category D or X affected 65.73% of admissions and were concentrated in a smaller subgroup, with 30.17% of admissions having ≥3 alerts. The interaction spectrum was dominated by nervous-system drugs and predictable additive CNS depression from sedative-analgesic combinations. After ICU process variables were added to the models, MV and medication count were the strongest correlates of both pDDI occurrence and burden, while associations with baseline characteristics were attenuated. The exploratory chart review identified documented clinical consequences in 32 of 50 sampled episodes and documented management actions in 29. These findings support the possible clinical relevance of some alerts rather than establishing that every database-defined D or X interaction caused harm. Therefore, the main information is that severe-pDDI alerts are common in this ICU setting, but they should be used as context-sensitive signals during clinical review rather than as alternative indicators for confirmed ADEs.

The reported prevalence of severe pDDIs varies significantly among adult, pediatric, and neonatal ICUs, driven by heterogeneity in study populations, design, case mix, exposure windows, interaction databases, severity definitions, and whether prescriptions or actual administrations are analyzed (Amkreutz et al., 2017; Lima et al., 2020; Chen et al., 2021; Costa et al., 2021; Baghaei et al., 2023; Han et al., 2025). Therefore, the observed prevalence here should not be interpreted as intrinsically higher or lower than any single published estimate. Restricting the analysis to confirmed medication administration records can reduce overestimation based solely on prescription data, although it cannot eliminate database-related heterogeneity (Bakker et al., 2022). The rate of 101.6 severe pDDIs per 1,000 medication administrations also conforms to the trend in the literature that the apparent burden falls when interactions are restricted to clinically relevant ones (Amkreutz et al., 2017; Bakker et al., 2021). In addition, the study-specific ordinal thresholds (0, 1–2, and ≥3 severe-pDDI alerts) describe the concentration of alert burden, rather than validated clinical risk stratification. Similar estimates under an alternative threshold support statistical robustness, but not clinical validation of the categories.

Medication use and the leading interaction pairs are expected to vary across countries and centers because ICU case mix, formularies, treatment protocols, and prescribing preferences differ. In this cohort, the use of alimentary-tract and metabolic drugs, respiratory drugs, ambroxol, and glutathione was prominent, which is consistent with the predominance of respiratory diagnoses and local emphasis on airway secretion management and organ support. While other ICU series have emphasized anti-infectives, nervous-system agents, cardiovascular drugs, or anticoagulants (Baniasadi et al., 2015; Dagdelen MS et al., 2021; Jafarova Demirkapu and Kara, 2021; Obeid and Karara, 2022; Arab A et al., 2023; Han et al., 2025). These differences make the exact ranking of drug pairs related to butorphanol, dexmedetomidine, and compound-methoxyphenamine more valuable locally, rather than universally applicable. However, at a broader level, the predominance of nervous-system agents and additive CNS depression is consistent with recurring ICU patterns, because sedatives and analgesics are frequently co-administered during analgosedation and ventilatory support, which is also in accordance with contemporary practice guidelines recommendations (Boncyk et al., 2024; Lewis K et al., 2025).

The distinction between a potential interaction alert and a clinically meaningful interaction is particularly important for the leading sedative-analgesic pairs. Combinations such as butorphanol-dexmedetomidine and butorphanol-midazolam can produce expected additive pharmacodynamic effects and may be intentionally co-prescribed to achieve analgesia, sedation, procedural tolerance, or ventilator synchrony (Ding et al., 2022; Riera et al., 2022; Jiang HY et al., 2023; Boncyk et al., 2024; Özgan et al., 2024; Lewis K et al., 2025). Lexi-Interact category D recommends consideration of therapy modification, whereas category X recommends avoiding the combination; neither category alone confirms an adverse outcome in an individual patient. The results of chart-review provide a preliminary clinical context, but documentation of respiratory depression, hypotension, or excessive sedation cannot establish temporal or causal attribution, and undocumented events may have been missed. Similarly, the noscapine-chlorphenamine alert in category X was largely formulation-driven because both ingredients occurred within compound methoxyphenamine products. Therefore, clinical interpretation should consider indication, dose, timing, duration of overlap, patient susceptibility, monitoring, and feasible alternatives. This approach is consistent with existing evidence, which shows that clinical adjudication substantially narrows the set of pDDIs requiring action and that selected high-risk interactions can nevertheless cause adverse events (Bakker et al., 2021; Klopotowska JE et al., 2024). Pooling D and X alerts was useful for estimating overall actionable alert burden, but it limits inference about category-specific predictors and consequences.

The associations of MV and medication count with pDDIs confirm established ICU risk patterns, rather than representing a new etiologic discovery (Dagdelen MS et al., 2021; Wang et al., 2022; Kayhan et al., 2025). Their value in the present analysis is operational: adding ICU process variables markedly improved model performance (binary-model AUC, 0.603–0.792; ordinal-model pseudo-R^2^, 0.041–0.390), and the associations remained stable after adjustment for ICU LOS and under an alternative burden definition. They also remained materially unchanged after the 29 readmission encounters were excluded. Although there is a correlation between MV and medication exposure, all VIFs were below 2, indicating no significant multicollinearity. MV should nevertheless be interpreted as a marker of treatment intensity and clinical complexity, not necessarily as an independent causal mechanism; mediation by medication exposure or other time-varying aspects of critical illness cannot be excluded. The inverse associations with diagnosis count and prior stroke were not consistently anticipated and may reflect residual confounding, coding practices, treatment limitations, or selection effects. These secondary findings require confirmation, particularly given the number of comparisons performed.

In the ordinal model, smoking history was associated with greater pDDI burden, but was not associated with the binary occurrence of any severe pDDI, so the finding remains exploratory. Tobacco smoke can induce CYP1A2, and abrupt smoking cessation during hospitalization may alter the exposure levels to susceptible drugs, providing a pharmacokinetic rationale for closer medication review in selected patients (Klomp et al., 2020; Barrangou-Poueys-Darlas et al., 2021). However, this mechanism is drug-specific and does not by itself explain a higher count of database alerts. Information on smoking intensity, timing of cessation, relevant CYP1A2 substrates, enzyme activity, and therapeutic drug monitoring was unavailable. The observed association should therefore not be interpreted as causal or used for risk stratification without external validation.

The principal practical implication lies in giving priority to conducting the review rather than treating every D or X alert identically. ICU-tailored decision support can reduce administration of high-risk combinations, and pharmacist-led interventions can reduce drug-related problems and improve regimen optimization (Ayhan et al., 2022; Bakker et al., 2024; Porubcova et al., 2024; Puxty et al., 2025). Locally frequent combinations, including butorphanol-dexmedetomidine, may be reasonable candidates for targeted monitoring, individualized dose titration, and review of safer alternatives, while recognizing that such combinations may have therapeutic value in selected settings (Ding et al., 2022; Wang et al., 2026). Alert priorities should be adapted to regional prescribing patterns and should incorporate clinical adjudication, patient-specific vulnerability, and documented management options. This strategy is more likely to reduce alert fatigue and focus resources on preventable or insufficiently monitored risk than blanket avoidance based solely on database category.

The use of confirmed administration data, staged modeling, sensitivity analyses, and an exploratory chart review strengthened the description of local alert burden. Several limitations nevertheless qualify the interpretations. First, the single-center retrospective design, local formulary, and regional prescribing practices restrict generalizability of specific drugs and interaction-pair findings. Second, the detection relied on one drug interaction database, categories D and X were pooled, and the ordinal burden thresholds were study-specific. Third, the chart review included only 50 episodes and depended on routine documentation; it could neither capture all consequences nor determine causality. Fourth, residual confounding and chance findings remain possible despite multivariable adjustment and sensitivity analyses, particularly for secondary associations. ICU readmissions constituted 2.48% of encounters; the primary models did not account for within-patient correlation, although exclusion of readmission encounters yielded materially similar results. Finally, the available smoking variable lacked exposure detail. Prospective multicenter studies using standardized clinical adjudication, category-specific analyses, and validated burden measures are needed to distinguish expected and manageable pharmacodynamic interactions from pDDIs that lead to preventable patient harm.

## 5 Conclusion

In conclusion, severe pDDIs (D + X) were frequent in this single-center ICU cohort, affecting nearly two-thirds of admissions. These interactions were dominated by additive PD CNS depression involving common sedative–analgesic combinations; database classification alone did not establish clinical harm. Mechanical ventilation and medication count were the principal independent correlates of both severe pDDI occurrence and burden, highlighting that treatment intensity rather than baseline characteristics primarily drives severe-pDDI exposure. Targeted, context-aware medication review and pharmacist-integrated workflows may therefore be useful in similar settings, but the specific drug pairs and exploratory burden thresholds require multicenter validation and should not be generalized across healthcare systems.

## Data Availability

The original contributions presented in the study are included in the article/Supplementary Material, further inquiries can be directed to the corresponding authors.
