# Supplementary material for: Incidence, burden, and associated factors of severe potential drug–drug interactions in intensive care unit patients: a real-world retrospective study
Source: Front Pharmacol. 2026 Sep 24;17:1920401. doi: 10.3389/fphar.2026.1920401 (PMC13645780; doi:10.3389/fphar.2026.1920401)
Supplement: Supplementary file 1 [file Table1.docx]

Supplementary Material

Table S1. ICU admissions and pDDI event counts by Lexi-Interact risk category

| **Risk rating** | **Number of** admissions | **Number of pDDIs** |
| --- | --- | --- |
| X | 429 | 1023 |
| D | 637 | 1681 |
| C | 1016 | 9624 |
| B | 639 | 1470 |
| A | 78 | 0 |

Table S2. Distribution of 213 severe-pDDI pairs

| **Drug pairs** | **Risk rating** | **Potential clinical consequences** | **Severity** | **Evidence** | **Mechanism of interaction** | **Frequency (total=2704)** |
| --- | --- | --- | --- | --- | --- | --- |
| Butorphanol - Dexmedetomidine | D | Increase CNS depressant effects | Major | Intermediate | PD | 335 |
| Butorphanol - Midazolam | D | Increase CNS depressant effects | Major | Intermediate | PD | 191 |
| Noscapine - Chlorphenamine | X | Increase adverse/toxic effects of Noscapine | Moderate | Intermediate-Low | PD | 176 |
| Butorphanol - Propofol | D | Increase CNS depressant effects | Major | Intermediate | PD | 122 |
| Dexmedetomidine - Midazolam | D | Increase CNS depressant effects | Moderate | Intermediate-Low | PD | 118 |
| Doxofylline - Aminophylline | X | Increase adverse/toxic effects of Doxofylline | Moderate | Intermediate-Low | PD | 91 |
| Dexmedetomidine - Chlorphenamine | D | Increase CNS depressant effects | Moderate | Intermediate-Low | PD | 84 |
| Noscapine - Dexmedetomidine | X | Increase adverse/toxic effects of Noscapine | Moderate | Intermediate-Low | PD | 83 |
| Dexmedetomidine - Propofol | D | Increase CNS depressant effects | Moderate | Intermediate-Low | PD | 82 |
| Esomeprazole - Clopidogrel | X | Decrease active metabolite exposure and antiplatelet effects of Clopidogrel | Major | Intermediate-Low | PK/PD | 70 |
| Spironolactone - Potassium Chloride | X | Increase hyperkalemic effects | Major | Intermediate | PD | 70 |
| Potassium Chloride - Chlorphenamine | X | Increase the risk of ulcers with potassium chloride | Moderate | Intermediate-Low | PD | 69 |
| Noscapine - Butorphanol | X | Increase adverse/toxic effects of Noscapine | Moderate | Intermediate-Low | PD | 68 |
| Butorphanol - Chlorphenamine | D | Increase CNS depressant effects | Major | Intermediate | PD | 68 |
| Ethanol - Butorphanol | X | Increase CNS depressant effects | Major | Intermediate | PD | 59 |
| Omeprazole - Clopidogrel | X | Decrease antiplatelet effects and active metabolite exposure of Clopidogrel | Major | Intermediate | PK/PD | 51 |
| Noscapine - Midazolam | X | Increase adverse/toxic effects of Noscapine | Moderate | Intermediate-Low | PD | 45 |
| Butorphanol - Valproic Acid | D | Increase CNS depressant effects | Major | Intermediate | PD | 41 |
| Bisoprolol - Dexmedetomidine | D | Increase rebound hypertensive and AV-blocking effects | Moderate | Intermediate-Low | PD | 38 |
| Linezolid - Norepinephrine | D | Increase hypertensive effects | Moderate | Intermediate | PD | 32 |
| Noscapine - Propofol | X | Increase adverse/toxic effects of Noscapine | Moderate | Intermediate-Low | PD | 30 |
| Butorphanol - Linezolid | X | Increase serotonin syndrome effects | Moderate | Intermediate-Low | PD | 29 |
| Dexmedetomidine - Valproic Acid | D | Increase CNS depressant effects | Moderate | Intermediate-Low | PD | 29 |
| Noscapine - Ethanol | X | Increase adverse/toxic effects of Noscapine | Moderate | Intermediate-Low | PD | 27 |
| Fluconazole - Midazolam | D | Increase serum concentrations of Midazolam | Moderate | Highest | PK | 23 |
| Ginkgo Biloba - Aspirin | D | Increase anticoagulant effects of Salicylates | Moderate | Intermediate-Low | PD | 23 |
| Meropenem - Valproic Acid | D | Decrease serum concentrations of Valproic Acid and Derivatives | Major | Intermediate | PK | 22 |
| Remifentanil - Dexmedetomidine | D | Increase CNS depressant effects | Major | Intermediate | PD | 22 |
| Remifentanil - Propofol | D | Increase CNS depressant effects | Major | Intermediate | PD | 18 |
| Voriconazole - Midazolam | D | Increase serum concentrations of Midazolam | Major | Highest | PK | 17 |
| Cefuroxime - Esomeprazole | D | Decrease absorption of Cefuroxime | Moderate | Intermediate | PD | 16 |
| Cefuroxime - Sodium Bicarbonate | D | Decrease serum concentrations of Cefuroxime | Moderate | Intermediate | PK | 15 |
| Remifentanil - Midazolam | D | Increase CNS depressant effects | Major | Intermediate | PD | 15 |
| Cefuroxime - Omeprazole | D | Decrease absorption of Cefuroxime | Moderate | Intermediate | PD | 14 |
| Butorphanol - Remifentanil | X | Decrease analgesic effects of Opioid Agonists | Major | Intermediate | PD | 13 |
| Dopamine - Linezolid | D | Increase hypertensive effects of Dopamine | Major | Intermediate-Low | PD | 13 |
| Linezolid - Dopamine | D | Increase hypertensive effects of Sympathomimetics | Moderate | Intermediate | PD | 13 |
| Itraconazole - Omeprazole | D | Increase or decrease the serum concentration of itraconazole | Major | Intermediate | PK | 12 |
| Propofol - Valproic Acid | D | Increase therapeutic effects of Propofol | Moderate | Intermediate-Low | PD | 12 |
| Itraconazole - Sodium Bicarbonate | D | Decrease serum concentrations of Itraconazole | Major | Intermediate | PK | 11 |
| Propofol - Amiodarone | D | Increase QTc-prolonging effects | Major | Intermediate-Low | PD | 11 |
| Ethanol - Remifentanil | X | Increase CNS depressant effects | Major | Intermediate | PD | 10 |
| Amiodarone - Digoxin | D | Increase serum concentrations of Digoxin | Major | Highest | PK | 10 |
| Amiodarone - Fluconazole | D | Increase QTc-prolonging effects | Major | Intermediate-Low | PK/PD | 10 |
| Linezolid - Dobutamine | D | Increase hypertensive effects of Sympathomimetics | Moderate | Intermediate | PD | 10 |
| Potassium Chloride - Hyoscyamine | X | Increase ulcerogenic effects of Potassium Chloride | Moderate | Intermediate-Low | PD | 9 |
| Butorphanol - Levetiracetam | D | Increase CNS depressant effects | Major | Intermediate | PD | 9 |
| Itraconazole - Esomeprazole | D | Increase or decrease the serum concentration of itraconazole | Major | Intermediate | PK | 9 |
| Metoprolol - Dexmedetomidine | D | Increase rebound hypertensive effects and AV-blocking effects | Moderate | Intermediate-Low | PD | 9 |
| Calcium Gluconate - Ceftriaxone | D | Increase adverse/toxic effects of Ceftriaxone | Major | Intermediate-Low | PD | 8 |
| Cefuroxime - Ranitidine | D | Decrease absorption of Cefuroxime | Moderate | Intermediate | PD | 8 |
| Dapagliflozin - Insulin Regular | D | Increase hypoglycemic effects of Insulin | Moderate | Intermediate-Low | PD | 8 |
| Noscapine - Valproic Acid | X | Increase adverse/toxic effects of Noscapine | Moderate | Intermediate-Low | PD | 7 |
| Prazosin - Urapidil | X | Increase antihypertensive effects of Alpha1-Blockers | Moderate | Intermediate-Low | PD | 7 |
| Esmolol - Dexmedetomidine | D | Increase rebound hypertensive effects and AV-blocking effects | Moderate | Intermediate-Low | PD | 7 |
| Pyridoxine - Levodopa | D | Decrease therapeutic effects of Levodopa-Foslevodopa | Moderate | Intermediate | PD | 7 |
| Ritonavir - Midazolam | X | Increase serum concentrations of Midazolam | Major | Highest | PK | 6 |
| Voriconazole - Amiodarone | X | Increase QTc-prolonging effects of Voriconazole | Major | Intermediate | PK/PD | 6 |
| Butorphanol - Nalmefene | D | Decrease therapeutic effects of Opioid Agonists | Major | Intermediate-Low | PD | 6 |
| Dexmedetomidine - Levetiracetam | D | Increase CNS depressant effects of Dexmedetomidine | Moderate | Intermediate-Low | PD | 6 |
| Methylprednisolone - Baricitinib | D | Increase immunosuppressive effects of Baricitinib | Moderate | Intermediate-Low | PD | 6 |
| Repaglinide - Clopidogrel | D | Increase serum concentrations of Repaglinide | Major | Intermediate | PK | 6 |
| Amiodarone - Levofloxacin | X | Increase QTc-prolonging effects of Amiodarone | Major | Intermediate-Low | PD | 5 |
| Nirmatrelvir and Ritonavir - Midazolam | X | Increase serum concentrations of Midazolam | Major | Highest | PK | 5 |
| Noscapine - Remifentanil | X | Increase adverse/toxic effects of Noscapine | Moderate | Intermediate-Low | PD | 5 |
| Acarbose - Insulin Glargine | D | Increase hypoglycemic effects of Insulin | Moderate | Intermediate-Low | PD | 5 |
| Cefuroxime - Pantoprazole | D | Decrease absorption of Cefuroxime | Moderate | Intermediate | PD | 5 |
| Dapagliflozin - Insulin Glargine | D | Increase hypoglycemic effects of Insulin | Moderate | Intermediate-Low | PD | 5 |
| Fluconazole - Clopidogrel | D | Decrease active metabolite exposure of Clopidogrel | Major | Intermediate | PK | 5 |
| Hyoscyamine - Sodium Bicarbonate | D | Decrease serum concentrations of Hyoscyamine | Minor | Intermediate-Low | PK | 5 |
| Remifentanil - Chlorphenamine | D | Increase CNS depressant effects | Major | Intermediate | PD | 5 |
| Moxifloxacin - Amiodarone | X | Increase QTc-prolonging effects | Major | Intermediate-Low | PD | 4 |
| Rivaroxaban - Nadroparin | X | Increase anticoagulant effects | Major | Intermediate-Low | PD | 4 |
| Acarbose - Insulin Regular | D | Increase hypoglycemic effects of Insulin | Moderate | Intermediate-Low | PD | 4 |
| Enoxaparin - Nadroparin | D | Increase anticoagulant effects | Moderate | Intermediate-Low | PD | 4 |
| Levothyroxine - Calcium Lactate | D | Decrease therapeutic effects of Thyroid Products | Moderate | Intermediate | PD | 4 |
| Sodium Zirconium Cyclosilicate - Clopidogrel | D | Decrease active metabolite exposure of Clopidogrel | Minor | Intermediate | PD | 4 |
| Ticagrelor - Aspirin | D | Aspirin may increase or decrease therapeutic effects of Ticagrelor | Major | Intermediate-Low | PD | 4 |
| Vancomycin - Colistin | D | Increase nephrotoxic effects of Colistimethate | Major | Intermediate-Low | PD | 4 |
| Metformin - Ethanol | X | Increase adverse/toxic effects of Metformin | Major | Intermediate | PD | 3 |
| Metronidazole - Carbocisteine | X | Increase adverse/toxic effects of products Containing Ethanol | Moderate | Lowest | PD | 3 |
| Metronidazole - Ethanol | X | Increase adverse/toxic effects of Alcohol (Ethyl) | Moderate | Lowest | PD | 3 |
| Voriconazole - Tamsulosin | X | Increase serum concentrations of Tamsulosin | Moderate | Intermediate | PK | 3 |
| Cetirizine - Dexmedetomidine | D | Increase CNS depressant effects | Moderate | Intermediate-Low | PD | 3 |
| Dexmedetomidine - Perphenazine | D | Increase CNS depressant effects | Moderate | Intermediate-Low | PD | 3 |
| Imipenem and Cilastatin - Valproic Acid | D | Decrease serum concentrations of Valproic Acid and Derivatives | Major | Intermediate | PK | 3 |
| Linezolid - Metaraminol | D | Increase hypertensive effects of Sympathomimetics | Moderate | Intermediate | PD | 3 |
| Remifentanil - Valproic Acid | D | Increase CNS depressant effects | Major | Intermediate | PD | 3 |
| Ritonavir - Clopidogrel | D | Reduce the antiplatelet effect of clopidogrel and the exposure level of its active metabolites | Moderate | Intermediate | PK/PD | 3 |
| Rosuvastatin - Febuxostat | D | Increase serum concentrations of Rosuvastatin | Moderate | Intermediate | PK | 3 |
| Doxazosin - Urapidil | X | Increase antihypertensive effects of Alpha1-Blockers | Moderate | Intermediate-Low | PD | 2 |
| Ethanol - Cetirizine | X | Increase sedative effects of Cetirizine | Moderate | Intermediate-Low | PD | 2 |
| Ethanol - Levocetirizine | X | Increase sedative effects of Levocetirizine | Moderate | Intermediate-Low | PD | 2 |
| Itraconazole - Tamsulosin | X | Increase serum concentrations of Tamsulosin | Moderate | Intermediate | PK | 2 |
| Levodopa - Ethanol | X | Increase serum concentrations of Levodopa | Moderate | Intermediate-Low | PK | 2 |
| Midazolam - Itraconazole | X | Increase serum concentrations of Midazolam | Major | Intermediate | PK | 2 |
| Noscapine - Levetiracetam | X | Increase adverse/toxic effects of Noscapine | Moderate | Intermediate-Low | PD | 2 |
| Noscapine - Perphenazine | X | Increase adverse/toxic effects of Noscapine | Moderate | Intermediate-Low | PD | 2 |
| Potassium Chloride - Atropine | X | Increase ulcerogenic effects of Potassium Chloride | Moderate | Intermediate-Low | PD | 2 |
| Potassium Chloride - Perphenazine | X | Increase ulcerogenic effects of Potassium Chloride | Moderate | Intermediate-Low | PD | 2 |
| Propafenone - Amiodarone | X | Increase QTc-prolonging effects of Propafenone | Major | Intermediate-Low | PD | 2 |
| Voriconazole - Fluconazole | X | Increase QTc-prolonging effects of Voriconazole | Moderate | Intermediate | PK/PD | 2 |
| Calcium Chloride - Ceftriaxone | D | Increase adverse/toxic effects of Ceftriaxone | Major | Intermediate-Low | PD | 2 |
| Cefuroxime - Lansoprazole | D | Decrease absorption of Cefuroxime | Moderate | Intermediate | PD | 2 |
| Dapagliflozin - Insulin Degludec | D | Increase hypoglycemic effects of Insulin | Moderate | Intermediate-Low | PD | 2 |
| Diltiazem - Midazolam | D | Increase serum concentrations of Midazolam | Moderate | Highest | PK | 2 |
| Ganciclovir - Imipenem and Cilastatin | D | Increase adverse/toxic effects of Imipenem | Moderate | Intermediate-Low | PD | 2 |
| Indobufen - Nadroparin | D | Increase anticoagulant effects of Heparins | Major | Intermediate-Low | PD | 2 |
| Itraconazole - Amiodarone | D | Increase serum concentrations of Amiodarone | Major | Intermediate | PK | 2 |
| Itraconazole - Atorvastatin | D | Increase serum concentrations of Atorvastatin | Moderate | Highest | PK | 2 |
| Itraconazole - Digoxin | D | Increase serum concentrations of Cardiac Glycosides | Moderate | Highest | PK | 2 |
| Methylprednisolone - Atracurium | D | Increase adverse neuromuscular effects of Corticosteroids | Major | Highest | PD | 2 |
| Nirmatrelvir and Ritonavir - Atorvastatin | D | Increase serum concentrations of Atorvastatin | Moderate | Intermediate | PK | 2 |
| Perphenazine - Norepinephrine | D | Increase arrhythmogenic effects of Antipsychotic Agents | Major | Lowest | PD | 2 |
| Propafenone - Digoxin | D | Increase serum concentrations of Cardiac Glycosides | Moderate | Highest | PK | 2 |
| Remifentanil - Linezolid | D | Increase adverse effects/toxicity of monoamine oxidase inhibitors and remifentanil | Moderate | Intermediate-Low | PD | 2 |
| Ritonavir - Atorvastatin | D | Increase serum concentrations of Atorvastatin | Moderate | Intermediate | PK | 2 |
| Torasemide - Celecoxib | D | Decrease diuretic effects of Torasemide and increase nephrotoxic effects of Celecoxib | Moderate | Highest | PD | 2 |
| Voriconazole - Ritonavir | D | Decrease or increase serum concentrations of Voriconazole | Major | Intermediate-Low | PK | 2 |
| Butorphanol - Pethidine Hydrochloride | X | Decrease analgesic effects of Opioid Agonists | Major | Intermediate | PD | 1 |
| Butorphanol - Remifentanil | X | Decrease analgesic effects of Opioid Agonists | Major | Intermediate | PD | 1 |
| Calcium Acetate - Calcium Gluconate | X | Increase adverse/toxic effects of Calcium Acetate | Moderate | Intermediate-Low | PD | 1 |
| Calcium Acetate - Calcium Lactate | X | Increase adverse/toxic effects of Calcium Acetate | Moderate | Intermediate-Low | PD | 1 |
| Ciclosporin - Atorvastatin | X | Increase serum concentrations of Atorvastatin | Major | Intermediate | PK | 1 |
| Clopidogrel - Ticagrelor | X | Increase antiplatelet effects of Ticagrelor | Major | Intermediate-Low | PD | 1 |
| Domperidone - Fluconazole | X | Increase QTc-prolonging effects of Fluconazole and Increase serum concentrations of Domperidone | Major | Intermediate | PK/PD | 1 |
| Domperidone - Levofloxacin | X | Increase QTc-prolonging effects of Domperidone | Moderate | Intermediate-Low | PD | 1 |
| Domperidone - Moxifloxacin | X | Increase QTc-prolonging effects of Domperidone | Moderate | Intermediate-Low | PD | 1 |
| Domperidone - Propofol | X | Increase QTc-prolonging effects of Domperidone | Moderate | Intermediate-Low | PD | 1 |
| Doxazosin - Phentolamine | X | Increase antihypertensive effects of Alpha1-Blockers | Moderate | Intermediate-Low | PD | 1 |
| Doxazosin - Tamsulosin | X | Increase antihypertensive effects of Alpha1-Blockers | Moderate | Intermediate-Low | PD | 1 |
| Flunarizine - Butorphanol | X | Increase CNS depressant effects of Flunarizine | Moderate | Intermediate-Low | PD | 1 |
| Flunarizine - Dexmedetomidine | X | Increase CNS depressant effects of Flunarizine | Moderate | Intermediate-Low | PD | 1 |
| Itraconazole - Domperidone | X | Increase serum concentrations of Domperidone | Major | Highest | PK | 1 |
| Itraconazole - Tolvaptan | X | Increase serum concentrations of Tolvaptan | Major | Intermediate | PK | 1 |
| Landiolol - Digoxin | X | Increase bradycardic effects of Landiolol | Moderate | Intermediate-Low | PD | 1 |
| Linezolid - Levodopa | X | Increase adverse/toxic effects of Linezolid | Major | Intermediate | PD | 1 |
| Metronidazole - Digoxin | X | Increase adverse/toxic effects of Products Containing Ethanol | Moderate | Lowest | PD | 1 |
| Metronidazole - Prednisone | X | Increase adverse/toxic effects of Products Containing Ethanol | Moderate | Lowest | PD | 1 |
| Metronidazole - Ritonavir | X | Increase adverse/toxic effects of Products Containing Ethanol | Moderate | Lowest | PD | 1 |
| Metronidazole - Sulfamethoxazole and Trimethoprim | X | Increase adverse/toxic effects of Products Containing Propylene Glycol | Moderate | Lowest | PD | 1 |
| Milnacipran - Linezolid | X | Increase serotonergic effects of Linezolid | Major | Intermediate | PD | 1 |
| Nirmatrelvir and Ritonavir - Rivaroxaban | X | Increase serum concentrations of Rivaroxaban | Major | High-Intermediate | PK | 1 |
| Nirmatrelvir and Ritonavir - Amiodarone | X | Increase serum concentrations of Amiodarone | Major | Intermediate | PK | 1 |
| Noscapine - Clonazepam | X | Increase adverse/toxic effects of Noscapine | Moderate | Intermediate-Low | PD | 1 |
| Noscapine - Estazolam | X | Increase adverse/toxic effects of Noscapine | Moderate | Intermediate-Low | PD | 1 |
| Noscapine - Fentanyl | X | Increase adverse/toxic effects of Noscapine | Moderate | Intermediate-Low | PD | 1 |
| Noscapine - Gabapentin | X | Increase adverse/toxic effects of Noscapine | Moderate | Intermediate-Low | PD | 1 |
| Phentolamine - Urapidil | X | Increase antihypertensive effects of Alpha1-Blockers | Moderate | Intermediate-Low | PD | 1 |
| Potassium Chloride - Flupentixol and Melitracen | X | Increase ulcerogenic effects of Potassium Chloride | Moderate | Intermediate-Low | PD | 1 |
| Potassium Chloride - Trihexyphenidyl | X | Increase ulcerogenic effects of Potassium Chloride | Moderate | Intermediate-Low | PD | 1 |
| Ritonavir - Tamsulosin | X | Increase serum concentrations of Tamsulosin | Moderate | Intermediate | PK | 1 |
| Ritonavir - Amiodarone | X | Increase serum concentrations of Amiodarone | Major | Intermediate | PK | 1 |
| Ritonavir - Propafenone | X | Increase serum concentrations of Propafenone | Major | Intermediate-Low | PK | 1 |
| Rivaroxaban - Heparin | X | Increase anticoagulant effects of Rivaroxaban | Major | Intermediate-Low | PD | 1 |
| Spironolactone - Ciclosporin | X | Increase hyperkalemic effects of Cyclosporine | Moderate | Intermediate-Low | PD | 1 |
| Tamsulosin - Urapidil | X | Increase antihypertensive effects of Alpha1-Blockers | Moderate | Intermediate-Low | PD | 1 |
| Acarbose - Gliclazide | D | Increase hypoglycemic effects of Sulfonylureas | Moderate | Intermediate-Low | PD | 1 |
| Amikacin - Vancomycin | D | Increase nephrotoxic and neurotoxic effects of Aminoglycosides | Moderate | Intermediate-Low | PD | 1 |
| Amiodarone - Colchicine | D | Increase serum concentrations of Colchicine | Major | Intermediate | PK | 1 |
| Atorvastatin - Daptomycin | D | Increase adverse/toxic effects of Daptomycin | Major | Intermediate-Low | PD | 1 |
| Azithromycin - Amiodarone | D | Increase QTc-prolonging effects of Azithromycin | Major | Intermediate-Low | PD | 1 |
| Betamethasone - Atracurium | D | Increase adverse neuromuscular effects of Betamethasone | Major | Highest | PD | 1 |
| Butorphanol - Eszopiclone | D | Increase CNS depressant effects of Opioid Agonists | Major | Intermediate | PD | 1 |
| Butorphanol - Flupentixol and Melitracen | D | Increase CNS depressant effects of Opioid Agonists | Major | Intermediate | PD | 1 |
| Butorphanol - Perphenazine | D | Increase CNS depressant effects of Opioid Agonists | Major | Intermediate | PD | 1 |
| Butorphanol - Promethazine | D | Increase CNS depressant effects of Opioid Agonists | Major | Intermediate | PD | 1 |
| Calcium Acetate - Ceftriaxone | D | Increase adverse/toxic effects of Ceftriaxone | Major | Intermediate-Low | PD | 1 |
| Carvedilol - Dexmedetomidine | D | Increase rebound hypertensive effects of Alpha2-Agonists and AV-blocking effects of Beta-Blockers | Moderate | Intermediate-Low | PD | 1 |
| Cefuroxime - Ilaprazole | D | Decrease absorption of Cefuroxime | Moderate | Intermediate | PD | 1 |
| Cetirizine - Chlorphenamine | D | Increase CNS depressant effects | Moderate | Intermediate-Low | PD | 1 |
| Cetirizine - Midazolam | D | Increase CNS depressant effects | Moderate | Intermediate-Low | PD | 1 |
| Cetirizine - Valproic Acid | D | Increase CNS depressant effects | Moderate | Intermediate-Low | PD | 1 |
| Dexmedetomidine - Eszopiclone | D | Increase CNS depressant effects and the adverse/toxic effects of Eszopiclone | Moderate | Intermediate-Low | PD | 1 |
| Dexmedetomidine - Eszopiclone | D | Increase CNS depressant effects of Dexmedetomidine | Moderate | Intermediate-Low | PD | 1 |
| Fentanyl - Chlorphenamine | D | Increase CNS depressant effects of Opioid Agonists | Major | Intermediate | PD | 1 |
| Fentanyl - Dexmedetomidine | D | Increase CNS depressant effects of Opioid Agonists | Major | Intermediate | PD | 1 |
| Flupentixol and Melitracen - Norepinephrine | D | Increase vasopressor effects of Alpha-/Beta-Agonists | Moderate | Intermediate-Low | PD | 1 |
| Heparin - Fondaparinux Sodium | D | Increase anticoagulant effects | Moderate | Intermediate-Low | PD | 1 |
| Heparin - Enoxaparin | D | Increase anticoagulant effects | Moderate | Intermediate-Low | PD | 1 |
| Hydrocortisone - Atracurium | D | Increase adverse neuromuscular effects of Corticosteroids | Major | Highest | PD | 1 |
| Indobufen - Enoxaparin | D | Increase anticoagulant effects of Heparins | Major | Intermediate-Low | PD | 1 |
| Itraconazole - Nifedipine | D | Increase serum concentrations of Nifedipine | Moderate | Intermediate | PK | 1 |
| Itraconazole - Pantoprazole | D | Increase or decrease serum concentrations of Itraconazole | Major | Intermediate | PK | 1 |
| Itraconazole - Ranitidine | D | Increase or decrease serum concentrations of Itraconazole | Major | Intermediate | PK | 1 |
| Linezolid - Epinephrine | D | Increase hypertensive effects of Sympathomimetics | Moderate | Intermediate | PD | 1 |
| Methotrexate - Celecoxib | D | Increase increase serum concentrations of Methotrexate | Major | Intermediate | PK | 1 |
| Methotrexate - Esomeprazole | D | Increase serum concentrations of Methotrexate | Moderate | Intermediate-Low | PK | 1 |
| Methotrexate - Ethanol | D | Increase hepatotoxic effects of Methotrexate | Moderate | Intermediate-Low | PD | 1 |
| Midazolam - Eszopiclone | D | Increase CNS depressant effects and adverse/toxic effects of Eszopiclone | Moderate | Intermediate-Low | PD | 1 |
| Midazolam - Promethazine | D | Increase CNS depressant effects of Promethazine | Moderate | Intermediate-Low | PD | 1 |
| Miglitol - Insulin Glargine | D | Increase hypoglycemic effects of Insulin | Moderate | Intermediate-Low | PD | 1 |
| Miglitol - Insulin Regular | D | Increase hypoglycemic effects of Insulin | Moderate | Intermediate-Low | PD | 1 |
| Moxifloxacin - Magnesium Chloride | D | Decrease serum concentrations of Quinolones | Moderate | Highest | PK | 1 |
| Omeprazole - Cilostazol | D | Decrease serum concentrations of Cilostazol and increase active metabolite exposure of Cilostazol | Moderate | Intermediate | PK/PD | 1 |
| Perphenazine - Dopamine | D | Increase arrhythmogenic effects of Phenothiazines | Major | Lowest | PD | 1 |
| Pethidine Hydrochloride - Midazolam | D | Increase CNS depressant effects of Opioid Agonists | Major | Intermediate | PD | 1 |
| Pethidine Hydrochloride - Promethazine | D | Increase CNS depressant effects of Opioid Agonists | Major | Intermediate | PD | 1 |
| Pethidine Hydrochloride - Valproic Acid | D | Increase CNS depressant effects of Opioid Agonists | Major | Intermediate | PD | 1 |
| Promazine - Norepinephrine | D | Increase arrhythmogenic effects of Phenothiazines | Major | Lowest | PD | 1 |
| Propofol - Fentanyl | D | Increase CNS depressant effects of Fentanyl | Major | Intermediate-Low | PD | 1 |
| Ritonavir - Itraconazole | D | Increase serum concentrations of Itraconazole | Moderate | Intermediate | PK | 1 |
| Roxadustat - Calcium Acetate | D | Decrease serum concentrations of Roxadustat | Moderate | Intermediate | PK | 1 |
| Roxadustat - Calcium Gluconate | D | Decrease serum concentrations of Roxadustat | Moderate | Intermediate | PK | 1 |
| Sevelamer - Roxadustat | D | Decrease serum concentrations of Roxadustat | Moderate | Intermediate | PK | 1 |
| Sitagliptin - Insulin Glargine | D | Increase hypoglycemic effects of Insulin | Moderate | Intermediate-Low | PD | 1 |
| Sitagliptin - Insulin Regular | D | Increase hypoglycemic effects of Insulin | Moderate | Intermediate-Low | PD | 1 |
| Torasemide - Ibuprofen | D | Decrease diuretic effects of Torasemide and increase nephrotoxic effects of Ibuprofen | Moderate | Highest | PD | 1 |
| Valproic Acid - Promethazine | D | Increase CNS depressant effects of Promethazine | Moderate | Intermediate-Low | PD | 1 |
| Verapamil - Midazolam | D | Increase serum concentrations of Midazolam | Moderate | Highest | PK | 1 |
| Voriconazole - Fentanyl | D | Increase serum concentrations of Fentanyl | Major | Intermediate | PK | 1 |
| Voriconazole - Nirmatrelvir and Ritonavir | D | Decrease or increase serum concentrations of Voriconazole | Major | Intermediate-Low | PK | 1 |
| Warfarin - Amiodarone | D | Increases the anticoagulant effect and serum concentration of vitamin K | Major | Intermediate | PK/PD | 1 |

PD, pharmacodynamics; PK, pharmacokinetics; CNS, central nervous system; AV-blocking, Atrioventricular Block.

Table S3. Sensitivity analysis of severe pDDI burden using an alternative ordinal classification (0, 1, and ≥2 episodes)

| **Candidate factors** | **Secondary outcome** | | | | |  |
| --- | --- | --- | --- | --- | --- | --- |
|  | **Multivariate analysis** | | | | |  |
|  | **Model 1** | |  | **Model 2** | | |
|  | **OR (95%CI)** | **p value** |  | **OR (95%CI)** | **p value** | |
| **Baseline variables** |  |  |  |  |  | |
| **Age, years, median (IQR)** | 1.00(0.99-1.01) | 0.492 |  | 1.00(0.99-1.01) | 0.564 | |
| **Gender** (N, Male/Female) | 1.46(1.16-1.85) | 0.001 |  | 1.15(0.89-1.49) | 0.296 | |
| **BMI (kg/m²)** | 1.00(0.99-1.01) | 0.581 |  | 1.00(0.99-1.01) | 0.842 | |
| **APACHE II score** | 1.03(1.02-1.05) | ＜0.001 |  | 0.99(0.98-1.01) | 0.548 | |
| **Number of diagnoses** | 1.00(0.97-1.03) | 0.889 |  | 0.94(0.901-0.97) | ＜0.001 | |
| **Hypertension** | 1.12(0.89-1.41) | 0.350 |  | 1.05(0.81-1.35) | 0.727 | |
| **Diabetes** | 0.87(0.67-1.11) | 0.259 |  | 0.86(0.65-1.14) | 0.291 | |
| **CHD** | 1.27(1.00-1.62) | 0.054 |  | 1.04(0.80-1.37) | 0.756 | |
| **Stroke** | 0.82(0.65-1.03) | 0.107 |  | 0.72(0.55-0.94) | 0.015 | |
| **Smoking** | 1.41(0.97-2.07) | 0.068 |  | 1.53(1.01-2.32) | 0.044 | |
| **Drinking** | 0.80(0.52-1.24) | 0.320 |  | 0.71(0.44-1.15) | 0.164 | |
| **Process variable** |  |  |  |  |  | |
| **MV** (N, Yes/No) |  |  |  | 2.35(1.82-3.05) | ＜0.001 | |
| **Number of ICU medications** |  |  |  | 1.17(1.14-1.19) | ＜0.001 | |

**Note:** **Secondary outcome:** Severe pDDI burden categorized into 0, 1, and ≥2 episodes, analyzed via ordinal logistic regression (proportional odds model). Model 1 adjusted for baseline variables available at admission (age, sex, BMI, APACHE II score, diagnosis count, and comorbidities). Model 2 additionally adjusted for ICU process variables (ICU medication count and mechanical ventilation). Females and the absence of corresponding comorbidities/events (e.g., absence of hypertension, diabetes, etc.) served as the reference groups for binary variables. The P-values for the parallel lines test in Model 1 and Model 2 (secondary outcome) were 0.053 and 0.174, respectively (P > 0.05), confirming model adequacy.

CI, confidence interval; IQR, interquartile range; OR, odds ratio; pDDIs, potential drug–drug interactions; MV, Mechanical ventilation; CHD, Coronary heart disease; APACHE II score, Acute Physiology and Chronic Health Evaluation II score.

Table S4. Sensitivity analysis including ICU length of stay as an additional covariate for severe pDDI occurrence and burden

| **Candidate predictor** | **Primary outcome** | | | | |  | **Secondary outcome** | | |
| --- | --- | --- | --- | --- | --- | --- | --- | --- | --- |
|  | **Multivariate analysis** | | | | |  | **Multivariate analysis** | | |
|  |  | **Model 2** | |  | **Model 2** | | | |  |
|  |  | **OR (95%CI)** | **p value** |  | **OR (95%CI)** | | | **p value** |  |
| **Baseline variables** |  |  |  |  |  | | |  |  |
| **Age, years, median (IQR)** |  | 1.00(0.99-1.01) | 0.612 |  | 1.00(1.00-1.01) | | | 0.462 |  |
| **Gender** (N, Male/Female) |  | 1.19(0.88-1.60) | 0.259 |  | 1.20(0.93-1.54) | | | 0.164 |  |
| **BMI (kg/m²)** |  | 1.00(0.99-1.01) | 0.983 |  | 1.00(0.99-1.01) | | | 0.759 |  |
| **APACHE II score** |  | 1.00(0.98-1.02) | 0.843 |  | 0.99(0.97-1.01) | | | 0.284 |  |
| **Number of diagnoses** |  | 0.92(0.89-0.96) | ＜0.001 |  | 0.94(0.91-0.97) | | | ＜0.001 |  |
| **Hypertension** |  | 1.01(0.75-1.36) | 0.946 |  | 1.03(0.80-1.32) | | | 0.799 |  |
| **Diabetes** |  | 0.75(0.54-1.05) | 0.089 |  | 0.81(0.62-1.07) | | | 0.136 |  |
| **CHD** |  | 1.12(0.81-1.54) | 0.508 |  | 0.94(0.72-1.23) | | | 0.656 |  |
| **Stroke** |  | 0.69(0.51-0.95) | 0.021 |  | 0.70(0.54-0.91) | | | 0.008 |  |
| **Smoking** |  | 1.23(0.75-2.01) | 0.420 |  | 1.56(1.05-2.33) | | | 0.029 |  |
| **Drinking** |  | 0.83(0.47-1.48) | 0.535 |  | 0.64(0.40-1.02) | | | 0.058 |  |
| **Process variable** |  |  |  |  |  | | |  |  |
| **MV** (N, Yes/No) |  | 2.44(1.81-3.30) | ＜0.001 |  | 2.45(1.89-3.17) | | | ＜0.001 |  |
| **Number of ICU medications** |  | 1.15(1.11-1.19) | ＜0.001 |  | 1.17(1.14-1.20) | | | ＜0.001 |  |
| **LOS** |  | 1.01(0.98-1.04) | 0.615 |  | 1.00(0.98-1.02) | | | 0.748 |  |

**Note:** **Primary outcome:** Severe pDDI occurrence (yes/no) analyzed via binary logistic regression. **Secondary outcome:** Severe pDDI burden categorized into 0, 1–2, and ≥3 episodes, analyzed via ordinal logistic regression (proportional odds model). Model 1 adjusted for baseline variables available at admission (age, sex, BMI, APACHE II score, diagnosis count, and comorbidities). Model 2 additionally adjusted for ICU process variables (ICU medication count and mechanical ventilation). Females and the absence of corresponding comorbidities/events (e.g., absence of hypertension, diabetes, etc.) served as the reference groups for binary variables. The P-values for the parallel lines test in Model 1 and Model 2 (secondary outcome) were 0.219 and 0.505, respectively (P > 0.05), confirming model adequacy.

CI, confidence interval; IQR, interquartile range; OR, odds ratio; pDDIs, potential drug–drug interactions; MV, Mechanical ventilation; CHD, Coronary heart disease; APACHE II score, Acute Physiology and Chronic Health Evaluation II score; ICU LOS, Length of stay in the intensive care unit.

Table S5. Sensitivity analysis excluding ICU readmission encounters: factors associated with severe-pDDI occurrence and burden

| **Candidate predictor** | **Primary outcome** | | | | | | | **Secondary outcome** | | | |
| --- | --- | --- | --- | --- | --- | --- | --- | --- | --- | --- | --- |
|  | **Bivariate analysis** | | | **Multivariate analysis** | | | | **Multivariate analysis** | | | |
|  | **With ≥1 severe pDDIs (N=749)** | **Without severe pDDIs (N=392)** | ***p* value** | **Model 1** | | **Model 2** | | **Model 1** | | **Model 2** | |
|  |  |  |  | **OR (95%CI)** | **p value** | **OR (95%CI)** | **p value** | **OR(95%CI)** | **p value** | **OR(95%CI)** | **p value** |
| **-Baseline variables-** |  |  |  |  |  |  |  |  |  |  |  |
| **Age (years)^a^** | 74 (64-82) | 72 (60-82) | 0.248 | 1.00(0.99-1.01) | 0.409 | 1.00(0.99-1.01) | 0.575 | 1.00(1.00-1.01) | 0.444 | 1.00(0.99-1.01) | 0.471 |
| **Sex (N,** Male/Female**)** | 505/244 | 227/165 | 0.001 | 1.50(1.15-1.96) | 0.003 | 1.20(0.89-1.62) | 0.241 | 1.51(1.19-1.90) | 0.001 | 1.20(0.93-1.55) | 0.167 |
| **BMI (kg/m²)^a^** | 23.0(20.2-26.0) | 22.5(20.0-25.4) | 0.034 | 1.00(0.99-1.01) | 0.554 | 1.00(0.99-1.01) | 0.974 | 1.00(0.99-1.01) | 0.679 | 1.00(0.99-1.01) | 0.709 |
| **APACHE II score^b^** | 20.40±7.21 | 18.76±7.44 | ＜0.001 | 1.03(1.01-1.05) | ＜0.001 | 1.00(0.98-1.02) | 0.733 | 1.03(1.01-1.04) | 0.001 | 0.99(0.97-1.01) | 0.229 |
| **Number of disease diagnoses^b^** | 8.43±4.21 | 8.41±4.09 | 0.930 | 0.99(0.95-1.02) | 0.414 | 0.93(0.89-0.96) | ＜0.001 | 1.00(0.98-1.04) | 0.639 | 0.94(0.91-0.97) | ＜0.001 |
| **Hypertension** | 436 | 223 | 0.667 | 1.02(0.78-1.34) | 0.859 | 0.98(0.73-1.33) | 0.903 | 1.09(0.87-1.38) | 0.461 | 1.03(0.80-1.32) | 0.827 |
| **Diabetes** | 201 | 124 | 0.088 | 0.76(0.57-1.02) | 0.066 | 0.75(0.54-1.04) | 0.081 | 0.85(0.66-1.09) | 0.201 | 0.80(0.61-1.06) | 0.125 |
| **CHD** | 359 | 166 | 0.072 | 1.25(0.94-1.66) | 0.121 | 1.08(0.78-1.50) | 0.628 | 1.16(0.91-1.48) | 0.238 | 0.91(0.70-1.20) | 0.513 |
| **Stroke** | 252 | 141 | 0.433 | 0.81(0.61-1.06) | 0.124 | 0.68(0.50-0.93) | 0.016 | 0.79(0.62-1.00) | 0.053 | 0.67(0.52-0.87) | 0.003 |
| **Smoking** | 144 | 63 | 0.189 | 1.25(0.80-1.95) | 0.326 | 1.23(0.74-2.05) | 0.417 | 1.44(0.99-2.10) | 0.055 | 1.57(1.05-2.37) | 0.030 |
| **Drinking alcohol** | 92 | 46 | 0.787 | 0.81(0.49-1.36) | 0.430 | 0.77(0.43-1.39) | 0.388 | 0.72(0.47-1.12) | 0.150 | 0.60(0.37-0.96) | 0.034 |
| **-Process variable-** |  |  |  |  |  |  |  |  |  |  |  |
| **MV (N,** Yes/No**)** | 498/251 | 129/263 | ＜0.001 |  |  | 2.46(1.81-3.33) | ＜0.001 |  |  | 2.52(1.94-3.27) | ＜0.001 |
| **Number of ICU medications^a^** | 23 (18-30) | 17 (14-21) | ＜0.001 |  |  | 1.15(1.12-1.18) | ＜0.001 |  |  | 1.17(1.14-1.19) | ＜0.001 |

Note: Model 1 included baseline variables available at admission. Model 2 additionally included MV and ICU medication count. Candidate variables were selected based on clinical relevance rather than solely statistical significance from univariable analyses. Females and the absence of corresponding comorbidities/events (e.g., absence of hypertension, diabetes, etc.) served as the reference groups for binary variables. ORs for continuous variables are per one-unit increase. The P-values for the parallel lines test in Model 1 and Model 2 (secondary outcome) were 0.177 and 0.411, respectively (P > 0.05), confirming model adequacy. pDDIs, Potential drug–drug interactions; IQR, interquartile range; CI, confidence interval; OR, odd ratio; MV, Mechanical ventilation; CHD, Coronary heart disease; APACHE II score, Acute Physiology and Chronic Health Evaluation II score.

^a^ Mann–Whitney U test. **^b^** Parametric T-test. All of the others were done by using Chi Square test.

Table S6. Goodness-of-fit and predictive performance of regression models

| **Outcome** | **Model** | **Nagelkerke R²** | **Hosmer-Lemeshow P** | **AUC** | **Parallel lines test P** |
| --- | --- | --- | --- | --- | --- |
| **Primary outcome**  (Severe pDDI occurrence) | Binary Model 1 | 0.043 | 0.864 | 0.603 | / |
|  | Binary Model 2 | 0.313 | 0.370 | 0.792 | / |
| **Secondary outcome**  (Severe pDDI burden) | Ordinal Model 1 | 0.041 | / | / | 0.219 |
|  | Ordinal Model 2 | 0.390 | / | / | 0.505 |

Table S7. STROBE checklist for cohort studies

|  | Item No | Recommendation | Page No |
| --- | --- | --- | --- |
| **Title and abstract** | 1 | (*a*) Indicate the study’s design with a commonly used term in the title or the abstract | Page 1 (title); lines 22–26 |
|  |  | (*b*) Provide in the abstract an informative and balanced summary of what was done and what was found | Page 1-2, lines 18-48 |
| **Introduction** | | | |
| Background/rationale | 2 | Explain the scientific background and rationale for the investigation being reported | Page 2-3, lines 50-94 |
| Objectives | 3 | State specific objectives, including any prespecified hypotheses | Page 3, lines 91-94 |
| **Methods** | | | |
| Study design | 4 | Present key elements of study design early in the paper | Page 3, lines 97-100 |
| Setting | 5 | Describe the setting, locations, and relevant dates, including periods of recruitment, exposure, follow-up, and data collection | Page 3, lines 97–100; Pages 3–4, lines 111–128 |
| Participants | 6 | (*a*) Give the eligibility criteria, and the sources and methods of selection of participants. Describe methods of follow-up | Page 3, lines 101–109; Figure 1, Page 13, lines 553–556 |
|  |  | (*b*) For matched studies, give matching criteria and number of exposed and unexposed | Not applicable |
| Variables | 7 | Clearly define all outcomes, exposures, predictors, potential confounders, and effect modifiers. Give diagnostic criteria, if applicable | Pages 3–5, lines 111–158 and 173–196 |
| Data sources/ measurement | 8 | For each variable of interest, give sources of data and details of methods of assessment (measurement). Describe comparability of assessment methods if there is more than one group | Pages 3–5, lines 110–148 and 159–172 |
| Bias | 9 | Describe any efforts to address potential sources of bias | Pages 4–5, lines 124–128 and 1602–172; Page 10, lines 389–403 |
| Study size | 10 | Explain how the study size was arrived at | Page 5; lines 182-187 |
| Quantitative variables | 11 | Explain how quantitative variables were handled in the analyses. If applicable, describe which groupings were chosen and why | Page 4-5, lines 150-158 and 174-187 |
| Statistical methods | 12 | (*a*) Describe all statistical methods, including those used to control for confounding | Page 5, lines 174-196; Table 4; Page 17-18, lines 580-586 |
|  |  | (*b*) Describe any methods used to examine subgroups and interactions | No formal interaction analyss; subgroup descriptions: Page 7; lines 259-265 |
|  |  | (*c*) Explain how missing data were addressed | Page 5; lines 183-185 |
|  |  | (*d*) If applicable, explain how loss to follow-up was addressed | Not applicable |
|  |  | (*e*) Describe any sensitivity analyses | Page 4-5; lines 157-158 and 189-196; Page 7, lines 275-284; Supplementary Table S3-S5 |
| **Results** | | | |
| Participants | 13 | (a) Report numbers of individuals at each stage of study—eg numbers potentially eligible, examined for eligibility, confirmed eligible, included in the study, completing follow-up, and analysed | Page 3 and 5, lines 102-109 and 199-205; Figure 1, Page 13, lines 553-556 |
|  |  | (b) Give reasons for non-participation at each stage | Page 3; lines 102-109 |
|  |  | (c) Consider use of a flow diagram | Figure 1, Page 13; lines 553-556 |
| Descriptive data | 14 | (a) Give characteristics of study participants (eg demographic, clinical, social) and information on exposures and potential confounders | Page 5-6; lines 198-214; Table 1, Page 14-15, lines 567-570 |
|  |  | (b) Indicate number of participants with missing data for each variable of interest | Page 5, lines 182-187 |
|  |  | (c) Summarise follow-up time (eg, average and total amount) | Page 5-6; lines 200-205; Table 1, Page 14-15, lines 567-570 |
| Outcome data | 15 | Report numbers of outcome events or summary measures over time | Page 6-8, lines 215-295; Tables 1 and 3-5, Pages 14-19, lines 567–589 |
| Main results | 16 | (*a*) Give unadjusted estimates and, if applicable, confounder-adjusted estimates and their precision (eg, 95% confidence interval). Make clear which confounders were adjusted for and why they were included | Pages 6-7, lines 237-284; Table 4, Pages 17-18, lines 579-586 |
|  |  | (*b*) Report category boundaries when continuous variables were categorized | Pages 4, lines 150-158; Pages 7, lines 253-265 |
|  |  | (*c*) If relevant, consider translating estimates of relative risk into absolute risk for a meaningful time period | Not applicable |
| Other analyses | 17 | Report other analyses done—eg analyses of subgroups and interactions, and sensitivity analyses | Pages 7-8, lines 258-295; Supplementary Table S3-S6 |
| **Discussion** | | | |
| Key results | 18 | Summarise key results with reference to study objectives | Pages 8, lines297-310 |
| Limitations | 19 | Discuss limitations of the study, taking into account sources of potential bias or imprecision. Discuss both direction and magnitude of any potential bias | Pages 10, lines 389-403 |
| Interpretation | 20 | Give a cautious overall interpretation of results considering objectives, limitations, multiplicity of analyses, results from similar studies, and other relevant evidence | Pages 8-10, lines 311-388 and 405-413 |
| Generalisability | 21 | Discuss the generalisability (external validity) of the study results | Pages 8–10, lines 311–336 and 391–403 |
| **Other information** | | | |
| Funding | 22 | Give the source of funding and the role of the funders for the present study and, if applicable, for the original study on which the present article is based | Pages 11, lines 429-430 |
